# Gallbladder ulcer erosion into the cystic artery: a rare cause ofupper gastro-intestinal bleeding Case report

**DOI:** 10.1186/1749-7922-5-8

**Published:** 2010-03-12

**Authors:** Offir Ben-Ishay, Mouad Farraj, Pavel Shmulevsky, Benjamin Person, Yoram Shimon Kluger

**Affiliations:** 1 Department of General Surgery B, Rambam Health Care Campus, Haifa, Israel

## Abstract

Intra luminal gallbladder bleeding is a rare cause of hemobilia that results in upper gastro-intestinal bleeding. In this case report we present a patient who presented with melena and eventually was diagnosed as bleeding from an ulcer in the gallbladder which was induced by gallstones and eroded into the cystic artery. Surgery revealed perforation of gallbladder which was the result of a pressure sore induced by a second gallstone.

## Introduction

Bleeding into the biliary tree or Hemobilia is a rare cause for upper gastro-intestinal bleeding that was first described by Francis Glison in 1654 [1]. Most commonly hemobilia is the result of trauma or investigatory interventions but inflammation, vascular malformation, malignancy and coagulopathy were also described as potential causes of hemobilia. A gallbladder ulcer eroding into the cystic artery is very rare, and only a handful of case reports of this entity are reported in the literature. When diagnosed, angioembolization followed by cholecystectomy is the recommended treatment.

We present a patient who was admitted due to melena and eventually was diagnosed as having hemobilia resulting from bleeding into the lumen of the gallbladder due to erosion of the cystic artery by gallstones.

## Case Description

**Figure 1:**
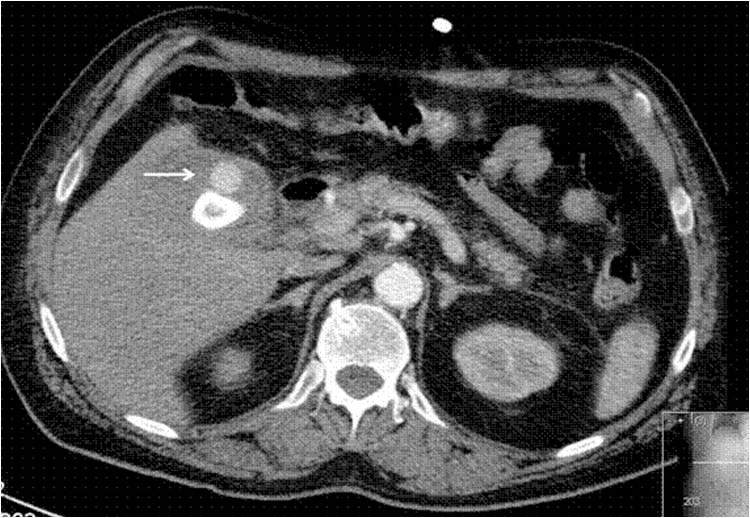
**Computerized Tomography showing active bleeding into the lumen of the gallbladder**.

A 68 year old man, with past history of ischemic heart disease, hypertension, hypercholesterolemia, fatty liver and gallstones presented to the Emergency Department complaining of colicky pain in the right upper abdominal quadrant and black tarry stools. On admission, the patient was hemodynamically stable with a heart rate of 80 beats per minute, a blood pressure of 140/80 mmHg, and Oxygen saturation of 98%. Physical examination revealed jaundice and marked tenderness in the right upper abdominal quadrant. Digital rectal examination revealed melena with no fresh blood. Laboratory results showed leukocytosis, slight elevation in total bilirubin (3.25 mg/dl), elevated gamma glutamyl transpeptidase (738 U/l) and alkaline phosphatase-B (391 U/l). Ultrasonography showed a gallbladder with features compatible with cholecystitis containing large stones. No dilatation of the intra and extra-hepatic bile ducts was noted. Upper endoscopy with a side view endoscope revealed blood coming through the duodenal papilla with no evident papillary pathology. Angiographic computerized tomography (Figure 1) revealed active bleeding into the lumen of the gallbladder that contained two large stones. Emergency surgery was elected rather than angioembolization due to clinical and laboratory indices of acute cholecystitis.

**Figure 2:**
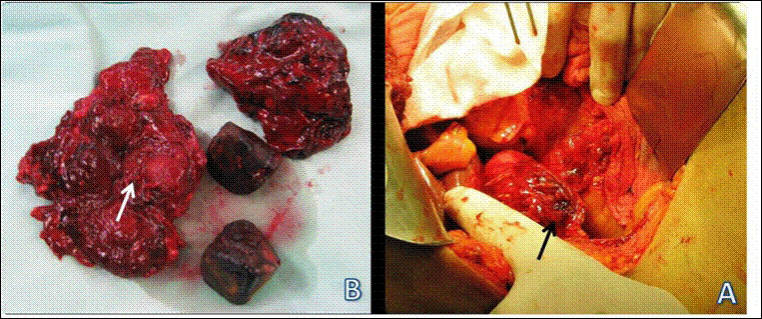
**A - Perforation of the gallbladder**. B - the respective ulcer leading to free perforation and the causing gallstones.

An open surgical exploration revealed the following findings: the omentum was adherent to the gallbladder and liver. The adjacent tissues were edematous and inflamed. The free wall of the gallbladder near the Hartmann's Pouch was perforated with blood clots obstructing the defect (Figure 2). Dissection of the gallbladder resulted in rupture of the gallbladder wall with massive bleeding from within its lumen. Control of the bleeding was achieved by a 5 minutes Pringle's maneuver that allowed the full dissection and removal of the gallbladder. Two large drains were left in the bed of the gallbladder and post operatively some bilious discharge was seen. The minor bile leak was managed conservatively with observation only and the discharge spontaneously ceased after several days.

On exploration of the resected specimen, two large gallstones were found, and a 0.5 cm ulcer was observed in the gallbladder wall.

Histopathologic examination was consistent with acute and chronic cholecystitis involving all layers of the organ that resulted in the formation of an ulcer with rupture of a pseudoaneurysm of the cystic artery.

The patient was discharged on the fourteenth post operative day; the drains were removed during the first postoperative outpatient clinic encounter and patient recovered uneventfully.

## Discussion and Conclusions

Spontaneous intra-cholecystic bleeding is a rare occurrence which was described in patients with gallstones [2] gallbladder malignancy [3] and patients receiving anticoagulant therapy [4]. Bleeding as a result of an ulcer eroding into an otherwise normal cystic artery or pseudoaneurysm of the cystic artery is rare [5-7]. When diagnosed, angioembolization of the bleeding cystic artery was suggested as the treatment of choice for bleeding control. In this report, we presented a patient who had large gallstones leading to the formation of a decubitus ulcer that eroded into the cystic artery with the formation of a pseudoaneurysm that ruptured and bled into the lumen of the gallbladder causing hemobilia with subsequent overt upper gastro-intestinal hemorrhage. A large gallbladder peroration, also presumed to be a result of a second decubitus ulcer was revealed during the surgical exploration.

Upper gastro-intestinal bleeding should be addressed promptly. If hemobilia is diagnosed and large stones in the gallbladder are detected, bleeding from a gallbladder ulcer should be ruled out. If angioembolization is elected, this should be followed immediately with surgery as the clinical set-up of bleeding due to gallstones might suggest a more complicated gallbladder disease than previously suspected.

## Decleration of competing interests

The authors declare that they have no competing interests.

## Authors' contributions

OBI - Study concept and design and drafted the manuscript, MF - Operating Surgeon, PS - Operating Surgeon, BP - Critical review study concept and design, YK - Critical review study concept and design. All authors read and approved the final manuscript

## Patient Consent

Written informed consent was obtained from the patient for publication of this case report and accompanying images. A copy of the written consent is available for review by the editor in chief of this journal.
